# Medical Reform in England

**Published:** 1845-08-01

**Authors:** 


*Medical Reform in England.*—Contemplated changes in the existing laws regulating the medical profession in England, have, of late, given rise to much and animated discussion. A bill was introduced sometime since, into the House of Commons, by Sir James Graham, upon which action has been deferred, in order to call out the sentiments of the Profession, and others, on the subject. Under the influence of these sentiments, the original bill has been once or twice amended. For the details and merits of the proposed reform, we must refer our readers to the British periodicals, a large portion of which for many months past has been occupied with discussions growing out of the contemplated alterations.

The following, which we quote from the *London News* of May 10th, refers to some later amendments of the Graham bill:

"PHYSIC AND SURGERY BILL.—SIR JAMES GRAHAM moved the committal, *pro forma*, of his Physic and Surgery Bill, in order to allow him to make amendments in it, which he fully explained to the house. He now proposes that there shall be three colleges—the College of Physicians, the College of Surgeons, and the College of General Practitioners. He proposes, likewise, to amend the Apothecaries Act, and to obliterate every vestige of that system of apprenticeship which he conceives to be injurious and degrading to the profession. He proposes also, to maintain the Council of Health in its present form, taking care that the general practitioners shall be represented in it, and that for this purpose two members of the College of General Practitioners shall be members of the Council of Health. The University of London is to be represented at the Council of Health, either by its Chancellor or its Vice-Chancellor. After much discussion the bill was comitted *pro forma*, and the proposed amendments introduced; the future stage having been moved by Sir JAMES GRAHAM for the 9th of June."

If we may credit the editor of the London Lancet, himself a member of Parliament, the bill, with all its emendations, creates almost universal dissatisfaction among the profession.

